# The neuropathology of fatal encephalomyelitis in human Borna virus infection

**DOI:** 10.1007/s00401-019-02047-3

**Published:** 2019-07-26

**Authors:** Friederike Liesche, Viktoria Ruf, Saida Zoubaa, Gwendolyn Kaletka, Marco Rosati, Dennis Rubbenstroth, Christiane Herden, Lutz Goehring, Silke Wunderlich, Miguel Frederic Wachter, Georg Rieder, Ines Lichtmannegger, Willibald Permanetter, Josef G. Heckmann, Klemens Angstwurm, Bernhard Neumann, Bruno Märkl, Stefan Haschka, Hans-Helmut Niller, Barbara Schmidt, Jonathan Jantsch, Christoph Brochhausen, Kore Schlottau, Arnt Ebinger, Bernhard Hemmer, Markus J. Riemenschneider, Jochen Herms, Martin Beer, Kaspar Matiasek, Jürgen Schlegel

**Affiliations:** 1 Department of Neuropathology, School of Medicine, Institute of Pathology, Technical University Munich, Trogerstraße 18, 81675 Munich, Germany; 2 Center for Neuropathology and Prion Research, Ludwig-Maximilians-Universitaet München, Munich, Germany; 3 Department of Neuropathology, University of Regensburg, Regensburg, Germany; 4 Section of Clinical and Comparative Neuropathology, Centre for Clinical Veterinary Medicine, Ludwig-Maximilians Universitaet München, Munich, Germany; 5 Institute of Diagnostic Virology, Friedrich-Loeffler-Institut, Greifswald-Insel Riems, Germany; 6 Institute of Veterinary Pathology, Justus Liebig University, Giessen, Germany; 7 Division of Medicine and Reproduction, Equine Hospital, Ludwig-Maximilians Universitaet München, Munich, Germany; 8 Department of Neurology, Klinikum rechts der Isar, School of Medicine, Technical University Munich, Munich, Germany; 9 Department of Pediatrics, Medical Faculty, Augsburg University, Augsburg, Germany; 10 Department of Neurology, Klinikum Traunstein, Traunstein, Germany; 11 Department of Pathology, Klinikum Traunstein, Traunstein, Germany; 12 Department of Pathology, Municipal Hospital Landshut, Landshut, Germany; 13 Department of Neurology, Municipal Hospital Landshut, Landshut, Germany; 14 Department of Neurology, Regensburg University Hospital, Regensburg, Germany; 15 Institute of Pathology, Medical Faculty, Augsburg University, Augsburg, Germany; 16 Department of Internal Medicine II, Municipal Hospital Landshut, Landshut, Germany; 17 Institute of Clinical Microbiology and Hygiene, Regensburg University Hospital, Regensburg, Germany; 18 Institute of Pathology, University of Regensburg, Regensburg, Germany; 19 Munich Cluster of Systems Neurology (SyNergy), Munich, Germany

**Keywords:** Virus, Bornavirus, Borna disease virus (BoDV-1), Zoonosis, Encephalitis

## Abstract

After many years of controversy, there is now recent and solid evidence that classical Borna disease virus 1 (BoDV-1) can infect humans. On the basis of six brain autopsies, we provide the first systematic overview on BoDV-1 tissue distribution and the lesion pattern in fatal BoDV-1-induced encephalitis. All brains revealed a non-purulent, lymphocytic sclerosing panencephalomyelitis with detection of BoDV-1-typical eosinophilic, spherical intranuclear Joest–Degen inclusion bodies. While the composition of histopathological changes was constant, the inflammatory distribution pattern varied interindividually, affecting predominantly the basal nuclei in two patients, hippocampus in one patient, whereas two patients showed a more diffuse distribution. By immunohistochemistry and RNA in situ hybridization, BoDV-1 was detected in all examined brain tissue samples. Furthermore, infection of the peripheral nervous system was observed. This study aims at raising awareness to human bornavirus encephalitis as differential diagnosis in lymphocytic sclerosing panencephalomyelitis. A higher attention to human BoDV-1 infection by health professionals may likely increase the detection of more cases and foster a clearer picture of the disease.

## Introduction

The classical Borna disease virus 1 (BoDV-1; species *Mammalian 1 orthobornavirus*) can lead to lethal infections in a broad range of mammals. The virus is endemically present in white-toothed bicolored shrews (*Crocidura leucodon*) [1, 7, 16], while horses and sheep are main accidental dead-end hosts [9]. Historically, a severe neurological disorder (Borna disease) due to an *encephalomyelitis lymphocytaria non-purulenta* with loss of neurons had been described for accidental hosts [18]. It is assumed that the virus enters the organism via the olfactory network and spreads through the limbic system to nearly all cortical areas, brainstem, cerebellum, and spinal cord [9]. In contrast to dead-end hosts, reservoir hosts feature a disseminated virus distribution to nearly every organ with shedding of infectious virus [24]. Other animals, including rats and bank voles, can be infected experimentally via different routes [10, 19]. Until recently, no report on confirmed human bornavirus infection existed. In 2015, the first human infections with another orthobornavirus (variegated squirrel 1 bornavirus [VSBV-1]*;* species *Mammalian 2 orthobornavirus*) were described, which resulted in lethal encephalitis of three breeders of variegated squirrels (*Sciurus variegatoides*). All three patients showed similar central nervous system (CNS) symptoms with lesions in cerebral cortex, basal nuclei, and brainstem, but without involvement of the spinal cord [17]. Subsequently, two more VSBV-1-induced human encephalitis cases occurred, whereof one was lethal [32, 33]. The first evidence for human infections with the “classical” BoDV-1 was published by two simultaneous reports just recently in 2018. A group of three recipients of solid organ transplants from the same donor developed severe encephalitis of preliminary unknown origin, which was later convincingly demonstrated to be BoDV-1-associated [28]. Furthermore, BoDV-1 infection led to the death of a previously healthy 25-year-old man [21]. Shortly afterwards, a report on a sporadic BoDV-1 infection with initial symptoms mimicking a Guillain–Barré syndrome was reported [5]. All five patients suffered a massive non-purulent encephalitis leading to death in four of the cases [5, 21, 28]. One of the deceased immunocompromised transplant recipients was autopsied and provided full insight into CNS and peripheral nerve pathology (patient 1). This incident triggered a broad research on post mortem cases with yet non-classified encephalitis. Our screening revealed autoptic material of five additional cases of fatal human BoDV-1. All patients had appeared completely healthy prior to the neurologic disease.

With these six cases, our study provides the first comprehensive description of the neuropathology of human BoDV-1 infection with focus on inflammatory lesions and virus distribution patterns at a systemic and cellular level.

## Materials and methods

This retrospective study was approved by the local ethical committee of the Technical University Munich. The families of patients 1, 2, and 6 gave informed consent to complete autopsy, whereas the families of patients 3, 4, and 5 agreed to brain autopsy only. For patients 1, 2, and 6, macroscopic evaluation of the thoracic and abdominal cavity, including evaluation of all organs, was conducted by two pathologists. For microscopic analysis, representative samples from peripheral organs, including heart, lung, liver, kidney, trachea, esophagus, stomach, intestine, and others were obtained. Brain dissection was carried out by two neuropathologists after routine formalin-fixation of the undissected brain. For microscopic analysis, representative samples from multiple selected regions of the brain and spinal cord were retrieved from following areas: frontal, parietal and occipital cortex, hippocampus, basal ganglia, mesencephalon, pons, medulla oblongata, cerebellum, as well as cervical, thoracic and lumbar parts of the spinal cord. From patients 1 and 6, peripheral nerves including the sciatic nerve and brachial plexus from patient 1, and sural and femoral nerve as well as brachial and sacral plexus from patient 6 were sampled in addition to the autonomic nerve branches attached to visceral organs and tissues. All tissue samples were further fixed in 10% buffered formalin and, after paraffin embedding, cut into 2 µm-thick sections. Subsequently, different stains were performed, including hematoxylin and eosin (H&E) stain, Grocott’s methenamine silver stain, and periodic acid-Schiff (PAS) stain. We conducted an established avidin–biotin complex technique for immunohistochemistry (IHC) using the monoclonal Bo18 antibody, which is directed against the nucleoprotein (N) of BoDV-1 [11], as described previously in animals [15, 35]. Furthermore, BoDV-1 RNA in situ hybridization (V-BoDV1-G targeting 2-969 of NC_001607-1:2236-3747, RNAscope^®^, Advanced Cell Diagnostics, Inc., USA) was performed as described previously [34]. For patients 1, 2, 3, and 5, infiltrating inflammatory cells were characterized by IHC using antibodies directed against CD3 (Cell Marque, The Netherlands; monoclonal, rabbit, dilution 1:500), CD20 (Dako Denmark A/S, Denmark; monoclonal, mouse, dilution 1:500), CD4 (Ventana Roche, Switzerland; monoclonal, rabbit, ready-to-use), CD8 (Ventana Roche, Switzerland; monoclonal, rabbit, ready-to-use), CD68 (Dako Denmark A/S, Denmark; monoclonal, mouse, dilution 1:15,000), CD138 (Cell Marque, The Netherlands; monoclonal, mouse, dilution 1:50), and Iba1 (abcam, USA; polyclonal, rabbit, dilution 1:500). These stainings were performed using a fully-automated staining system (Ventana BenchMark ULTRA; Ventana Medical Systems; Tucson; USA). Briefly, the slides were exposed to heat-induced epitope uncovering in pH 8.4 buffer at 95 °C for 40 min. After incubating with H_2_O_2_, the slides were incubated with the primary antibodies indicated above, followed by visualization using the 3,3′-diaminobenzidine-(DAB-)based OptiView DAB IHC Detection Kit (Ventana Medical Systems).

To characterize the cellular distribution of the infection, immunolabelling with Bo18 antibody was combined with cell markers Iba1 (abcam, USA; polyclonal rabbit, dilution 1:500), GFAP (Dako Denmark A/S, Denmark; rabbit polyclonal, dilution 1:500), S-100 (Dako Denmark A/S, Denmark; rabbit polyclonal, 1:500) and synaptophysin (Synaptic Systems, Germany; rabbit polyclonal, dilution 1:200) using an antibody removal agent (LinBlock, Linaris, Germany) and a green second chromogen (Histogreen^®^, Linaris, Germany).

In the same vein, RNA in situ hybridization of BoDV-1 RNA (V-BoDV1-G targeting 2-969 of NC_001607-1:2236-3747, RNAscope^®^, Advanced Cell Diagnostics, Inc., USA) was performed, followed by immunohistochemical counterstaining with glial fibrillary acidic protein (GFAP; Dako Denmark A/S, Denmark; monoclonal, mouse, dilution 1:500), S-100 (Agilent, USA; polyclonal, rabbit, dilution 1:6000), myelin basic protein (MBP; Cell Marque Corporation, USA; polyclonal, rabbit, ready-to-use, dilution 1:2), neurofilament (NFpan; Dako Denmark A/S, Denmark; monoclonal, mouse, dilution 1:200), Iba1 (abcam, USA; polyclonal, rabbit, dilution 1:500), CD68 (Dako Denmark A/S, Denmark; monoclonal, mouse, dilution 1:15.000) and CD3 (Cell Marque, The Netherlands; monoclonal, rabbit, dilution 1:500). BoDV-1-positive horse brain sections were used as controls. Lesions and immunohistochemical patterns were recorded and mapped for the individual patients.

For ultrastructural analyses, primary formalin-fixed brain samples from patient 6 were postfixed with Karnovsky-fixative (0.1 M cacodylate-buffer with 2.5% glutaraldehyde and 2% paraformaldehyde), followed by fixation in 1% osmium tetroxide at pH 7.3 for 2 h. Afterwards, they were dehydrated in graded ethanols, and embedded in the EMbed-812 epoxy resin (all reagents from Science Services, Germany; automated LYNX-tissue processor Leica, Germany). After 48 h of heat polymerisation at 60° C, semithin (0.8 µm) sections were cut and stained with toluidine blue and basic fuchsin. Light microscopy was used for selection of representative cells and ultrathin (80 nm) sections were cut with a diamond knife on a Reichert Ultracut-S ultramicrotome (Leica, Germany) and double stained with aqueous 2% uranyl acetate and lead citrate solutions for 10 min each. The sections were examined in a LEO912AB electron microscope (Zeiss, Germany) operating at 100 kV, equipped with a side-mounted CCD-camera (TRS, Lagerlechfeld / Germany) capable to record images with 2kx2k pixels. Documentation was done with the iTEM software (Olympus Soft Imaging Solutions GmbH, Germany).

For all six patients, the presence of BoDV-1 RNA in the brain was confirmed by reverse transcriptase quantitative polymerase chain reaction (RT-qPCR) and complete BoDV-1 genome sequencing approaches as described earlier [28].

## Results

### Clinical presentation

The six patients were all female and between 13 and 78 years of age (median 38 years). Five patients were apparently healthy prior to neurological disease, without any signs of immunodeficiency. One patient (patient 1) was therapeutically immunosuppressed after kidney transplantation [28].

Initially, four patients developed flu-like symptoms, including fever, followed by neurological signs, which progressed to loss of consciousness. One patient presented with right-sided weakness and unsteady gait. Death of the five previously healthy patients occurred between 2 and 6 weeks after admission to the hospital. The immunosuppressed patient 1 developed a severe axonal motor neuropathy with Guillain–Barré syndrome-like spread on post-transplant day 80. Symptoms progressed to tetraplegia and deficit of all cranial nerves, followed by persistent coma and death 14 weeks after initial symptoms. For detailed clinical presentation, see Table 1.

**Table 1:** Clinical presentation

	Patient 1	Patient 2	Patient 3	Patient 4	Patient 5	Patient 6
Age	74	21	13	17	78	55
Medical state prior to infection	Kidney transplantation due to end-stage diabetic nephropathy; subsequently immunosuppression	Healthy, no signs of immunosuppression	Healthy, no signs of immunosuppression	Healthy, no signs of immunosuppression	Healthy, no signs of immunosuppression	Healthy, no signs of immunosuppression
Initial symptoms	Axonal motor neuropathy with Guillain-Barré syndrome-like spread	Flu-like symptoms including fever	Flu-like symptoms including fever	Flu-like symptoms including fever	Right-sided weakness, unsteady gait, febrile	Fever, headache
Subsequent symptoms	Tetraplegia, deficit of all cranial nerves	Memory deficits, apathy, epileptic seizures	Slurred speech, ataxia, nystagmus, progressive somnolence, absent gag reflex	Headache, confusion	Epileptic seizure	Amnesic aphasia, word finding difficulties
Late symptoms	Persistent coma, vegetative neuropathy	Progressive loss of consciousness and brain stem reflexes	Progressive loss of consciousness, vegetative dysregulation with hypertonia and tachycardia	Progressive loss of consciousness	Progressive loss of consciousness, respiratory failure	Progressive loss of consciousness
Treatment	IVIG, plasma exchange	Antibiotics (doxycycline, ampicillin, ceftriaxon), antiviral (acyclovir), high-dose steroids, IVIG, plasma exchange	Antibiotics (doxycycline, ampicillin, ceftriaxon), antiviral (gancyclovir), high-dose corticosteroids	Antibiotics (ampicillin, sulbactam), antiviral (acyclovir), later high-dose steroids, plasmapheresis	Antibiotics (ampicillin, ceftriaxon), antiviral (acyclovir)	High-dose steroids, immunoadsorption
cMRI	Signs of diffuse supra- and infratentorial encephalitis with accentuation in the limbic system	Lesions in the right hippocampus without contrast enhancement progressing to extended lesions of the whole cortex, basal nuclei, thalamus, pons and medulla oblongata	Alterations with accen-tuation in the left caput nuclei caudate indicative for encephalitis	Cerebral and cerebellar edema	Increased signal intensity in the right temporal lobe	No specific changes
Time from initial symptoms to death	14 weeks	5 weeks	4 weeks	6 weeks	4 weeks	2 weeks

*IVIG* intravenously administered IgG-immunoglobulins, *cMRI* cranial magnetic resonance imaging

### Findings of the general organ autopsies

General autopsy of patient 1 revealed signs of multiple organ failure. Both autologous kidneys displayed high-grade atrophy with underlying mesangioproliferative glomerulonephritis. The transplanted kidney revealed no clear signs of rejection, but showed numerous nodular lymphocytic infiltrates, containing CD20- and CD3-positive cells, whereof the latter were composed of approximately equal proportions of CD4- and CD8-positive cells. Underlying diseases including atherosclerosis of aorta and coronary vessels plus myocardial hypertrophy and fibrosis as well as chronic pulmonary emphysema but no signs of a clinically suspected pneumonia were observed.

General autopsy of patient 2 revealed massive pulmonary edema and acute pulmonary congestion with consecutive acute biventricular cardiac dilation. Except for signs of shock, the other organs were without pathological findings. Likewise, in case of patient 6, no signs of inflammation were seen in the peripheral organs.

General autopsies were not conducted for the remaining patients, as the families gave consent to brain autopsy only.

### Macroscopic examination of the CNS

Macroscopically, the brain of patient 1 showed a considerable increase in volume with softened texture. Transtentorial as well as tonsillar herniation was observed on both sides. Coronal brain dissection revealed livid discoloration of the cerebral cortex with areas of an indefinite cortico-medullary junction. The ventricular system appeared constricted at full-length but without midline shift. Cerebellum, brain stem, and spinal cord were increased in volume, too. The brain of patient 2 appeared macroscopically unremarkable. In the brain of patient 3, the basal nuclei were softened on both sides. Globally, the brain of patient 4 appeared softened with mild edema. Incipient tonsillar herniation was noted. After coronal dissection, the cerebral and cerebellar tissue were of softened texture with accentuation of the cortical areas. The brain of patient 5 appeared edematous with discretely prominent cerebellar tonsils but without distinct tonsillar herniation. After coronal brain dissection, the ventricular system was slightly constricted but without any midline shift. Malacia of the hippocampal region was noted on both sides. The brain of patient 6 showed uncal herniation on the left side, but without considerable compression of the midbrain. Coronal sections of the cerebrum displayed slight edema of the subcortical white matter.

### Microscopic examination of the CNS and PNS

**Fig. 1:**
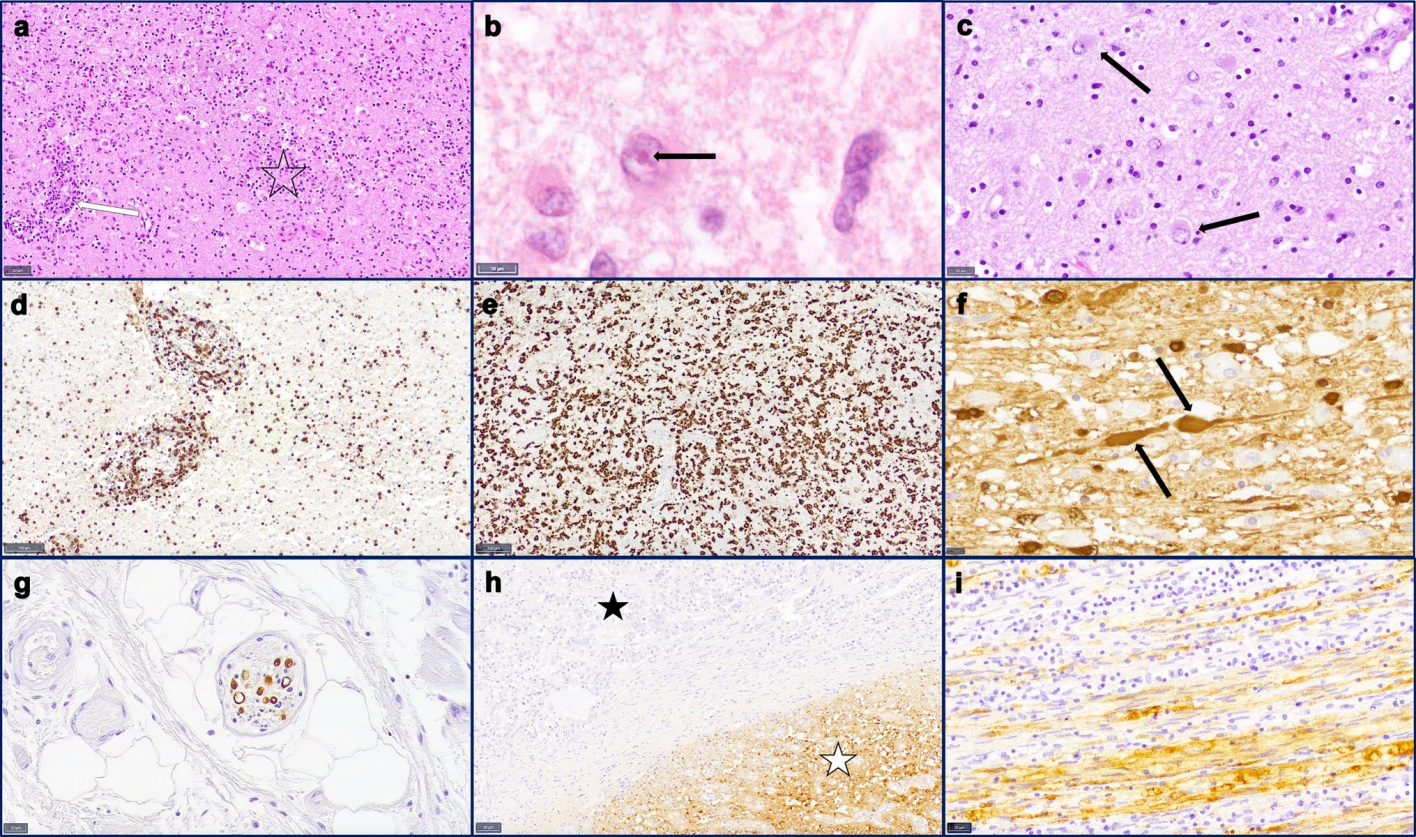
Microscopic changes in human Borna disease. **a** H&E shows perivascular accentuated (arrow) and diffuse distributed lymphocytic infiltrates, microglial nodules (star) and a distinct reactive astrogliosis Scale bar: 50 µm, basal nuclei, patient 1. **b** H&E reveals intranuclear, eosinophilic inclusions (arrows), known as Joest–Degen bodies in a reactive astrocyte. Scale bar: 10 µm, cortex, patient 2. **c** H&E shows distinct astrogliosis with remarkably enlarged reactive astrocytes exhibiting enlarged nuclei with thin and marginal chromatin and opaque-eosinophilic cytoplasm. Scale bar: 20 µm, cortex, patient 4. **d** CD3 immunohistochemistry shows the lymphocytic distribution with perivascular accentuation as well as diffuse parenchymal infiltration. Scale bar: 100 µm, hippocampal, patient 2. **e** Iba1 immunohistochemistry confirms strong activation of the microglia and macrophages. Scale bar: 100 µm, basal nuclei, patient 3. **f** Bo18 immunohistochemistry indicates virus infestation of neurons with distinct positivity of axons with nodular protuberance (arrows). Scale bar: 10 µm, pons, patient 2. **g** Virus protein detection by Bo18 immunohistochemistry in a small nerve in the surrounding tissue of the esophagus. Scale bar: 20 µm, patient 1. **h** Bo18 immunohistochemistry of the pituitary gland with strong positivity of the posterior pituitary (white star) but no detection of virus protein in the adenohypophysis (black star). Scale bar: 50 µm, pituitary gland, patient 2. **i** Virus protein detection by Bo18 immunohistochemistry in the sciatic nerve; dense lymphocytic infiltrates (arrow). Scale bar: 20 µm, sciatic nerve, patient 1

Cerebral tissue of all six patients exhibited a non-purulent panencephalomyelitis with edematous and spongy texture as well as perivasculary accentuated infiltration of lymphocytes (Fig. 1a). The number of diffusely infiltrating lymphocytes was highest in patient 1, followed by patients 4 and 5, whereas the remaining patients showed substantially lower numbers. Even though lymphocytic infiltrates were found in all examined samples, the inflammation was accentuated in the basal nuclei of patients 3 and 4 and hippocampal area of patient 5. In the CNS of patient 6, inflammation was mostly pronounced in the upper brain stem and subcortical nuclei. The brains of patients 1 and 2 showed a more global diffuse infiltration with no clear accentuation. Diffusely distributed macrophages, filled with eosinophilic, PAS-positive material, and plasma cells were observed most prominently in patient 1. Beyond formation of microglial nodules, all brains demonstrated distinct astrogliosis with remarkably enlarged reactive astrocytes exhibiting enlarged nuclei with thin and marginal chromatin and opaque-eosinophilic cytoplasm (Fig. 1c). In patient 1, global malacia with tremendous loss of neurons with remaining nuclear debris was observed. In patient 4, severe malacia was noted in the hippocampus and in the cortex of frontal and occipital lobe. The loss of neurons was less pronounced in the brains of the remaining patients. In all patients, neurons and astrocytes showed eosinophilic, spherical, intranuclear inclusions without clear halo, so-called Joest–Degen inclusion bodies (Fig. 1b).

The majority of infiltrating lymphocytes were CD3-positive (Fig. 1d), with more CD4-positive than CD8-positive cells. Furthermore, IHC revealed numerous CD68-positive macrophages and strong microglial (Fig. 1e) and glial activation, demonstrated by Iba1 and GFAP immunostaining.

Brain stems and spinal cords displayed inflammatory changes as well whereas the cerebella were milder affected. Here, a global, almost complete loss of Purkinje cells with Bergmann glia proliferation was observed in patients 1, 2, 4, and 6, which was considerably milder in patients 3 and 5.

Sections of peripheral nerves of patient 1 showed massive necrotizing lymphocytic neuritis (Fig. 1i) with CD4- and predominantly CD8-positive lymphocytes. Small nerves of peripheral organs in patients 1 and 2 appeared inconspicuous, though.

**Fig. 2:**
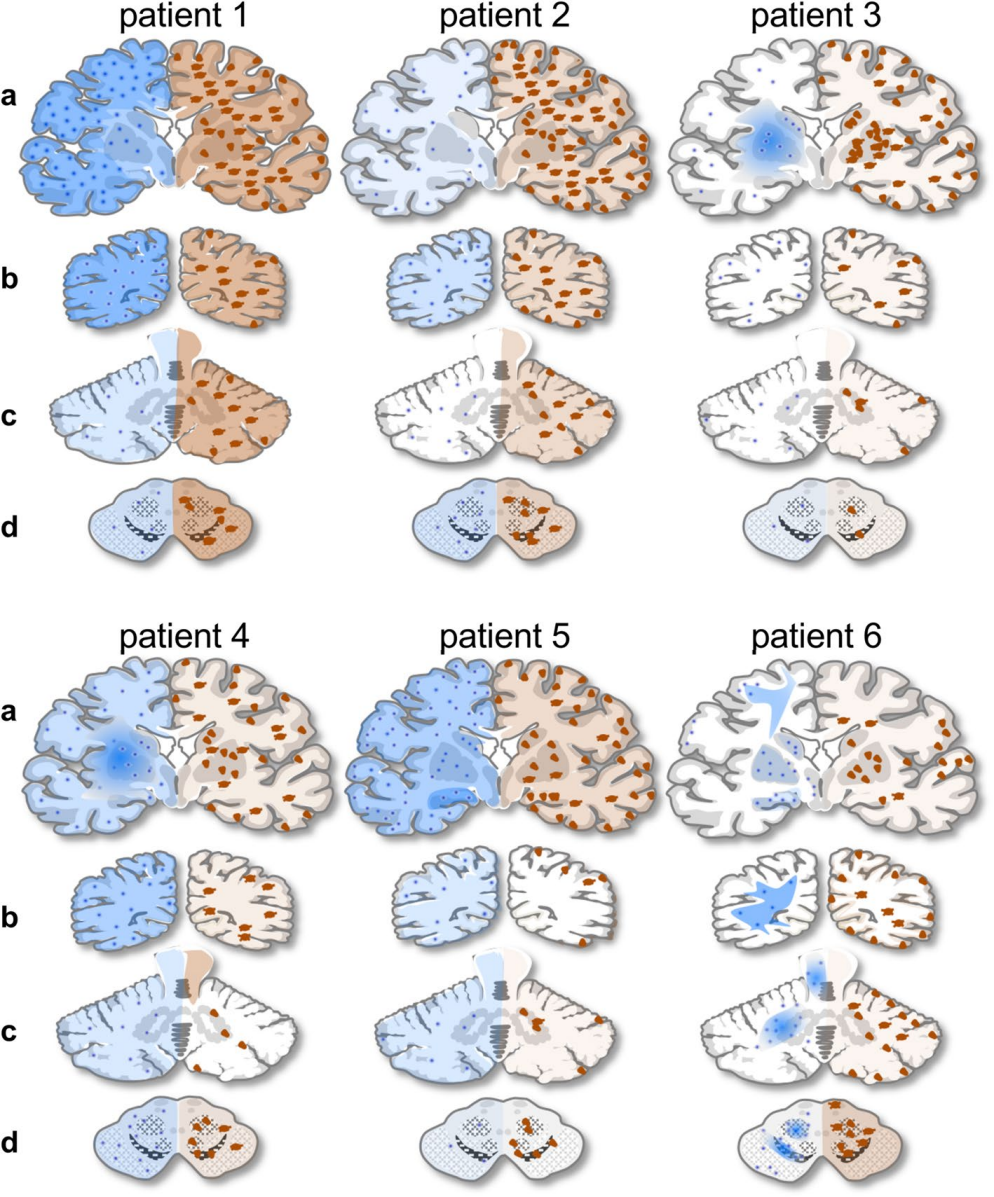
Interindividual differences of human BoDV-1 encephalitis. Schematic illustration of inflammatory changes (left side, blue) and virus antigen distribution (right side, brown) of all six patients with coronar sections including a level through the mammillary bodies with the basal nuclei (**a**), the occipital lobe (**b**), the cerebellum (**c**), and midbrain (**d**). Blue areas demonstrate diffuse parenchymal lymphohistiocytic infiltrates, while blue dots represent perivascular cuffs. Diffuse somatic and neuritic immunopositivity is illustrated by brown areas. Small groups of immunopositive neurons are demonstrated by brown triangles, groups of immunopositive astrocytes by brown star-like figures. Note the distinct enhancement of inflammation in the basal nuclei of patient 3

For local distribution of lymphocytic infiltration, microglial activation, edematous, and hypoxic changes, see Table 2 and Fig. 2.

**Table 2:** Tabular depiction of the local differences of the neuropathological changes in human BoDV-1 encephalitis

	Diffuse lymphocytic infiltrates						Perivascular lymphocytic cuffing						Microglial activation						Malacia						Edema					
	P1	P2	P3	P4	P5	P6	P1	P2	P3	P4	P5	P6	P1	P2	P3	P4	P5	P6	P1	P2	P3	P4	P5	P6	P1	P2	P3	P4	P5	P6
Frontal cortex	++	+	–	++	++	+	+++	+	+	+++	++	+	+++	++	–	++	+++	+	+++	+	–	+++	+	–	+++	+	–	++	++	++
Parietal cortex	+++	+	–	+	++	–	+++	+	++	+++	+	+	+++	+++	–	++	++	+	+++	+	–	+	–	–	+++	+	–	++	++	+
Occipital cortex	+++	++	–	++	+	+	+++	++	+	++	+	++	+++	+++	–	++	+	+++	+++	+	–	+++	–	+	+++	++	–	++	+	++
Striatum	++	+	++	+++	++	+	+++	+	+++	+++	+++	+++	+++	+	+++	+++	+++	+++	+++	+	++	+++	++	–	+++	+	++	+++	++	+
Thalamus	++	+	++	n.a.	+	+	+++	–	+++	n.a.	+++	+++	+++	+	+	n.a.	+	+++	+++	+	+	n.a.	–	–	+++	+	+	n.a.	+	+
Hippocampus	+	+	+	+++	++	++	++	+	+	+++	+	+++	+++	+	+	+++	+++	+++	+	+++	+	+++	+++	++	++	+	+	++	+++	+
Hypothalamus	+	+	++	n.a.	++	+	++	+	+++	n.a.	++	++	++	+	+++	n.a.	++	+++	++	+	+	n.a.	+	–	++	+	+	n.a.	++	–
Mesencephalon	+	+	+	+++	+	+++	++	+	++	+++	+	+++	+	+	+++	+++	+	+++	++	+	++	++	–	++	++	+	+	++	–	++
Pons	+	+	–	++	+	+++	++	++	++	+++	++	+++	++	++	–	++	++	+++	++	+	–	+	+	++	++	++	+	++	+	++
Medulla oblongata	+	+	+	++	+	++	+	++	+++	++	+	+++	++	++	+	++	–	+++	++	+	–	++	–	++	++	+	+	++	–	+
Cerebellum	+	–	+	+	+	+	–	++	++	++	+	+++	++	+	++	+	–	++	+	–	–	+	–	+	+	–	+	+	–	+

Patients 1–6 (P1–P6); +++: strong, ++: mild, +: weak, –: none, n.a.: not analyzed. The table shows local and interindividual differences in the intensity of the neuropathological changes

### Immunohistochemistry of BoDV-1 nucleoprotein and BoDV-1 RNA in situ hybridization

**Fig. 3:**
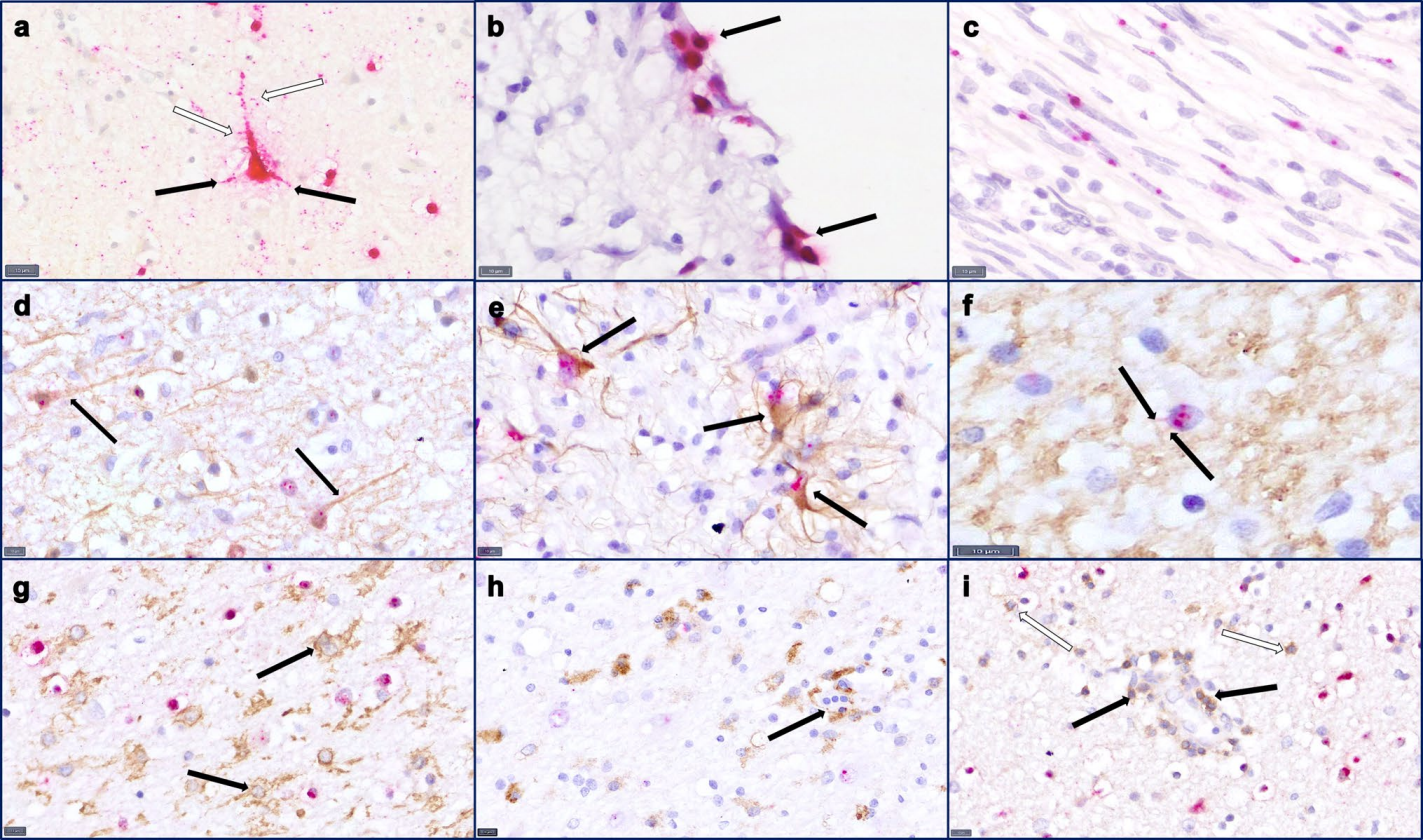
RNA in situ hybridization and co-stainings. **a** RNA in situ hybridization shows red signal for virus RNA in neurons and glial cells. A pyramidic cell shows virus RNA in dendrites (black arrows), including small dendritic branches (white arrows). Scale bar: 10 µm, cortical, patient 2. **b** RNA in situ hybridization indicates virus infestation of ependymal cells. Scale bar: 10 µm, patient 4. **c** RNA in situ hybridization detects BoDV1 in peripheral nerves. Scale bar: 10 µm, sciatic nerve, patient 1. Co-stainings of RNA in situ hybridization and immunohistochemical cellular markers show BoDV-1 RNA in NFpan-positive neurons (**d**, arrows), GFAP-positive reactive astrocytes (**e**, arrows) and MBP-positive oligodendrocytes (**f**, arrows point at MBP-positive processes). In contrast, Iba1-positive microglial cells (**g**, arrows), CD68-positive macrophages (**h**, arrow points at perivascular located macrophages) and CD3-positive lymphocytes (**i** black arrow points at perivascular located lymphocytes, white arrow at diffusely distributed lymphocytes) show no virus infestation. Scale bars: 10 µm; **d**, **g**, **i** cortical, patient 2, **e** hippocampal, patient 4, **f**, **h** cortical, patient 5

BoDV-1 nucleoprotein and RNA were detected in all examined CNS tissue samples including the spinal cord of all six patients, with less BoDV-1-positive cells detectable in the brain of patient 3 as compared to all other patients. Immunoreaction and RNA signal were present in neuronal cells, astrocytes, and oligodendrocytes. The neurons showed somatic as well as axonal and dendritic positivity (Figs. 1f, 3a).

Patient 1 showed a strong diffuse BoDV-1 distribution in all examined samples of cerebrum, cerebellum, and brain stem without clear focal accentuation. A uniform distribution of BoDV-1-positive cells was seen in patient 2, but with a smaller number of positive cells. Patients 3 and 4 showed a distinct accentuation of viral presence in the basal nuclei, primarily in neuronal, less in glial cells. In patient 4, samples of frontal, parietal, and occipital cortex and white matter showed a strong positivity in subcortical reactive astrocytes with only a small number of positive neurons and cortical glial cells. In patient 5, an accentuation of BoDV-1-positive neurons was noted in the hippocampal area on both sides. Interestingly, the occipital lobe showed a clearly lower number of BoDV-1-positive neurons and glial cells compared to other cortical and subcortical locations. In patient 6, the strongest immunoreaction was observed in the samples of the mesencephalon, cerebellum, and hippocampal region. Interestingly, the cortex of the occipital lobe showed a strong immunoreaction in the neurons as well as in the glial cells, whereas glial cells of the subcortical white matter were completely BoDV-1-negative. In all patients, the ependymal cells were partially BoDV-1-positive (Fig. 3b). For cerebral virus distribution, see also Fig. 2.

The pituitary gland of patient 2 showed a strong immunoreaction and RNA signal of the posterior pituitary compared to absence of BoDV-1 in the adenohypophysis (Fig. 1h) demonstrating the strong neurotropism of BoDV-1 in accidental dead-end hosts.

Furthermore, a positive immunoreaction and detection of BoDV-1 RNA were observed in all examined peripheral nerves of patient 1 (Figs. 1i, 3c). Diminutive nerves of visceral organs were BoDV-1-positive in both tested patients, including trachea and esophagus of patient 1 (Fig. 1g) and adrenal gland and thyroid gland of patient 2. No viral antigen or RNA was detected in the parenchyma and endothelial cells of visceral organs, though.

### Co-staining of BoDV-1 nucleoprotein and RNA with cellular markers

To determine the cell types infected by BoDV-1, co-staining for the BoDV-1 nucleoprotein with either GFAP, synaptophysin, or S-100 was performed. In the CNS, BoDV-1 antigen was detected in synaptophysin-positive neurons and GFAP-positive astrocytes. In the PNS, S-100-positive Schwann cells were strongly positive for BoDV-1 antigen.

These observations were corroborated by BoDV-1 RNA in situ hybridization combined with IHC. NFpan-positive neurons (Fig. 3d), as well as GFAP-positive reactive astrocytes (Fig. 3e) and MBP-positive oligodendrocytes (Fig. 3f) showed strong BoDV-1 RNA signal. No RNA signal was seen in microglial cells (Fig. 3g), macrophages (Fig. 3h) and lymphocytes (Fig. 3i). In the PNS, viral RNA was associated with NFpan-positive axons and S-100-positive Schwann cells.

### Electron microscopy

**Fig. 4:**
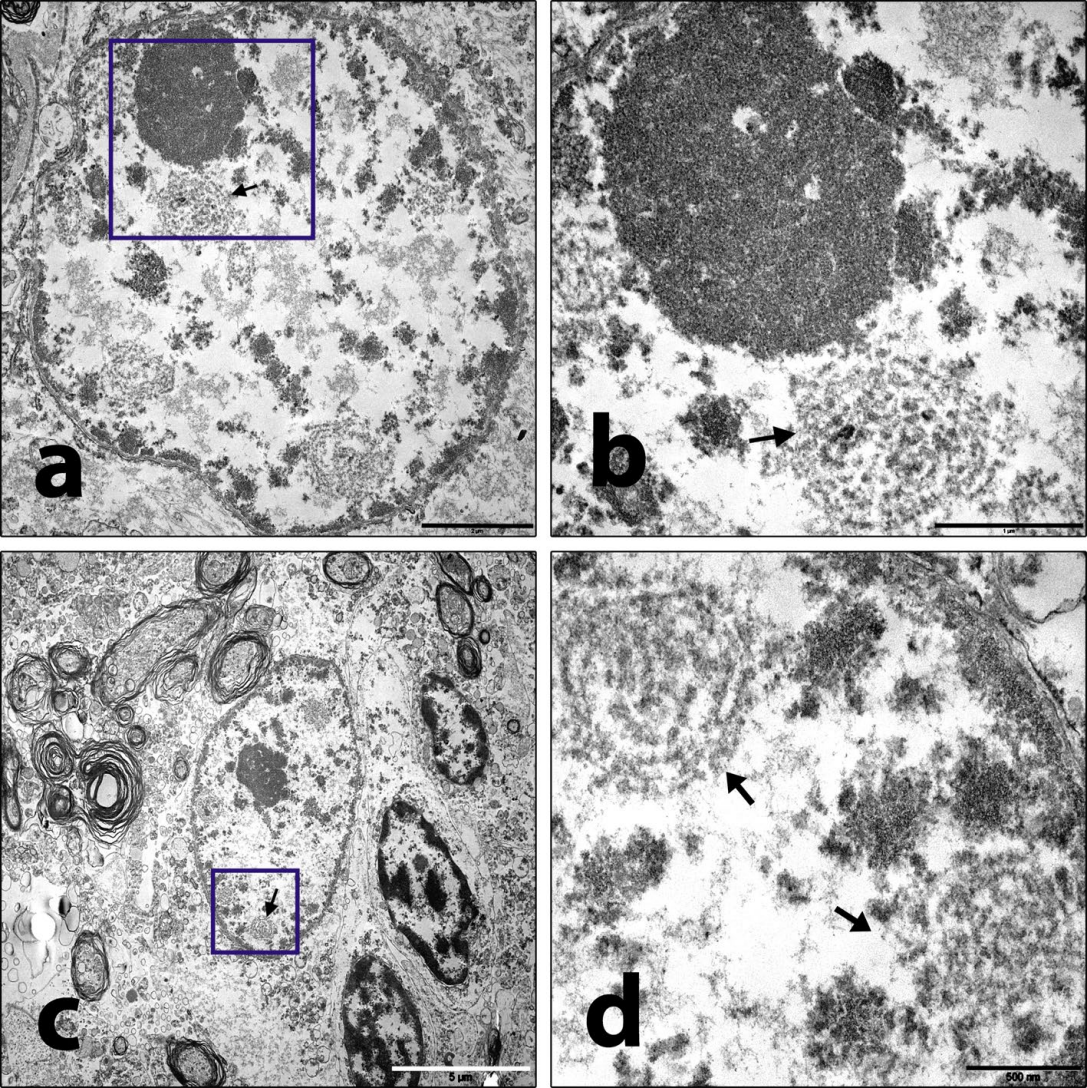
Electron microscopy of subcortical white matter of patient 6. **a**, **c** Low resolution shows astrocytic nuclei containing concentric structures (arrows). Scale bar **a** 2 µm (× 10,000), scale bar **c** 5 µm (× 5000)*.***b**, **d** High power views of **a** and **c** insets (areas within rectangles) display detailed architecture of viral replication centers (arrows). Scale bar **b** 1 µm (× 25,000), scale bar **d** 500 nm (× 40,000)

Transmission electron microscopy (TEM) of neocortical, subcortical, and brainstem specimens of patient 6 showed perivascular accentuated lymphomonocytic inflammation at low magnification. Intranuclear inclusions were predominantly seen in astrocytes and oligodendrocytes (Fig. 4a, c). Higher magnification of these inclusions revealed concentric complexes of membranous structures, the architecture characteristic of viral RNA replication centers (Fig. 4b, d).

## Discussion

Numerous descriptions of the neuropathological findings of BoDV-1 encephalitis in naturally infected animals have been published since 1911 when Joest and Degen gave the first report on Borna disease in horses [18]. Nevertheless, a detailed neuropathological depiction of BoDV-1 infection in humans is missing so far, as human bornavirus infections were confirmed only recently in VSBV-1-infected breeders and animal care takers of exotic squirrels [17, 32, 33] and in BoDV-1-infected patients, including three recipients of organ transplants from the same donor [5, 21, 28]. All patients had developed encephalitis and neurologic disease resembling Borna disease in horses and sheep, which had been fatal in most cases [5, 17, 21, 28, 32, 33]. One organ recipient was autopsied at our institute and gave for the first time deeper insights into human BoDV-1 encephalitis [28].

Through BoDV-1 screening of current post mortem cases with yet non-classified encephalitis, autoptic material of five additional sporadic cases of fatal BoDV-1 infection was detected. With a series of six cases, we are now able to give a first detailed and systematic description of the neuropathological findings in human BoDV-1 infection.

All brains featured similarities in their histopathological appearance, which can be summarized as lymphocytic sclerosing panencephalomyelitis with strong formation of microglial nodules. With co-staining of BoDV-1 RNA in situ hybridization and cellular markers, infected cell types including neurons, astrocytes, and oligodendrocytes were identified. In contrast, microglial cells as well as macrophages and lymphocytes were not infected. In neurons, BoDV-1 was detected in cell bodies as well as neuritic and dendritic processes. Furthermore, in all patients, the so-called Joest–Degen inclusion bodies were found. Due to their morphology, these intranuclear inclusions are accounted among Cowdry-type B inclusions [6, 18, 26].

Even if the histopathological aspects were consistent in all patients, they are not specific for bornavirus encephalitis, though, as other virus encephalitides exhibit similar appearances [4, 8]. Perivascularly accentuated lymphocytic infiltrates, together with glial and microglial activation is often found in arbovirus infections including tick-borne encephalitis [8]. Nevertheless, nuclear inclusion bodies as seen in all BoDV-1-infected patients have not been described for tick-borne encephalitis and might be a helpful distinctive criterion. It should be noted that Southern Germany, the home area of all patients of this study, is an endemic region for both, tick-borne encephalitis virus and BoDV-1 [14, 20]. Furthermore, the strong sclerosing aspect due to glial activation together with perivascular accentuated inflammatory infiltrates as seen in bornavirus encephalitis might resemble subacute sclerosing panencephalitis (SSPE) as secondary manifestation of measles virus infection [4]. Although the clinical aspects of SSPE usually differ from the described courses of BoDV-1 infection, bornaviruses should be considered as possible differential diagnoses.

In contrast to the constancy of the histopathological changes, interindividual differences regarding the distribution patterns of viral antigens and inflammatory infiltrates were observes. Two patients showed accentuation of lymphocytes in the basal nuclei, whereas in one patient, the hippocampal area and in another the upper brain stem were primarily affected. Two patients, however, showed a more uniform distribution including the grey as well as the white matter without clear local accentuation. It should be noted, however, that the described distribution patterns reflect the end-stage pathology after terminal disease. Nevertheless, accentuations might indicate primarily affected sites that may be targeted by diagnostic biopsy testing. All five previously healthy patients developed central neurological deficits early in the course of disease, whereas peripheral symptoms were not predominant. In contrast, the immunocompromised patient 1 developed initial symptoms mimicking Guillain–Barré syndrome, which is in line with BoDV-1 infection and inflammation observed in the PNS of this patient. Similar symptomology had been described for the second kidney recipient [28] and a recently published previously healthy BoDV-1-infected patient [5]*.* Furthermore, small nerves of peripheral organs of patients 1 and 2, including trachea and esophagus, were BoDV-1-positive. Infectious neuropathies have been described for other viruses, including hepatitis C virus and several herpesviruses, but are very rare incidents and occur only in a rather late course of the virus infections [2]. For patient 1, though, the neuritis was an early event, given the development of peripheral symptoms before any signs of CNS affection. Infection of the PNS has already been observed in experimentally BoDV-1-infected animals [19]. For human VSBV-1 infection, no PNS infection was described; the spinal cord showed no affection either [17, 32, 33]. Naturally BoDV-1-infected accidental animal hosts, including horses and sheep, rarely show signs of PNS infection [9]. In contrast, reservoir hosts, such as BoDV-1-infected bicolored white-toothed shrews or VSBV-1-infected squirrels, harbor the virus not only in central and peripheral nervous tissue, but also in non-neuronal cells including mesenchymal and epithelial cells of nearly every organ system, e.g., the respiratory and urinary tracts. However, bornavirus-induced inflammation is not observed in these hosts [7, 24, 27, 29]. Infectious virus can be detected in several excretions of BoDV-1-infected shrews, including saliva, urine, and feces [24], which is assumed to infect horses and possibly other accidental hosts via an olfactory route [23], followed by a spread to the limbic system [9]. This may explain the primary affection of these phylogenetically older areas of the CNS in horses [10, 22]. For humans, the route of entry has not been clarified, yet. Only for patient 1, the infected kidney transplant was confirmed as the source of infection, since retrospectively, the donor was retrospectively proven to be seropositive for BoDV-1, all three organ recipients suffered from bornavirus encephalitis, and identical viral sequences were found in the brains of both kidney recipients [28]. A significant accentuation of the limbic system indicating an olfactory route, as described for horses [10, 22] was observed only in patient 5, whereas patients 3 and 4 revealed a strong accentuation of the basal nuclei.

Interestingly, the course of disease was prolongated in patient 1 compared to the other patients (14 weeks compared to 2–6 weeks). The cytotoxicity in BoDV-1 infection is known to be mediated by BoDV-1-specific CD8-positive T lymphocytes [3, 13, 30]. Thus, therapeutic immunosuppression after kidney transplantation might have decelerated the course of disease in patient 1 and might give lead to therapeutic possibilities. T-lymphocyte depletion or suppression starting before or early after experimental BoDV-1 infection was demonstrated to prevent immunopathology in rodent models [12, 25, 31].

The strong interindividual differences lead to several intriguing questions including infections routes, dose of infectious virus, the impact of the duration of infection, susceptibility, and potential risk factors. Here, we present the first comprehensive description on the neuropathology of human victims of bornavirus encephalitis. Our report is based on only six autopsies since the first confirmed human BoDV-1 infections appeared just recently [5, 21, 28]. Our data aim to initiate a better understanding of the circumstances and pathobiology of human Borna disease that are clearly needed to identify so far undiagnosed cases and develop an epidemiological basis.

## Conclusion

On the basis of six brain autopsies, human BoDV-1 infection was shown to result in lymphocytic sclerosing panencephalomyelitis with detection of BoDV-1-typical eosinophilic, spherical, intranuclear Joest–Degen inclusion bodies. Bornavirus infection should be considered in cases matching these characteristics. While the composition of histopathological changes appears consistent, the lesion distribution pattern varies interindividually, which might indicate different routes of entry or individual immune status. Infection of the PNS may occur and results in clinical presentation resembling Guillain–Barré syndrome in some cases. The common and overlapping neuropathological signs we here describe will likely stimulate the identification of additional cases. These could then be used to gain a clearer depiction of locoregional manifestation patterns and to the identification of favorable locations for diagnostic biopsies.
